# Occult Orbital Injury with Dagger Fragment with Resulting Pneumocephalus

**DOI:** 10.1155/2018/5093417

**Published:** 2018-09-18

**Authors:** Lucía Jáñez-García, Enrique Mencía-Gutiérrez, Esperanza Gutiérrez-Díaz, Luis F. Moreno-García-Rubio, Laura Zarratea-Herreros, Álvaro Bengoa-González, Silvia Pérez-Trigo

**Affiliations:** Ophthalmology Department, 12 de Octubre Hospital, Complutense University, 28041 Madrid, Spain

## Abstract

Penetrating injuries of the cranium are relatively uncommon, only 0.4% of all head injuries. In patients with disturbed conscious level, an extensive examination should be performed in the emergency unit to rule out transorbital penetrating brain injury. A 25-year-old male was attacked with a dagger. He presented with ethylic intoxication and the physical examination demonstrated a small skin injury on the lateral canthus of the left eye with a large periocular hematoma which prevented eyelid opening. Cranial CT scan showed a metallic intraorbital foreign body consisting of a fragment of a dagger which perforated the eyeball, and penetrated through the superomedial wall of the orbit into the anterior cranial fossa. Reconstruction of the eyeball was performed and the fragment was removed. Orbital injuries with a knife in situ are very unusual. Early identification and removal of retained foreign bodies are essential.

## 1. Introduction

Traumatic eye injuries due to retained large foreign bodies are rare [1]. Eyelid or periocular wounds may be the only initial clinical sign of a penetrating orbitocranial foreign body [2]. Retained orbital dagger fragments are rare [3–11]; we present a case in which an unnoticed dagger fragment, only detected by image tests, caused eyeball perforation and orbital fracture with pneumocephalus. Transorbital penetrating brain injury with open globe is an unusual occurrence, representing about 0.4% of all head injuries [12]. We report a young male with an intraorbital fragment of a dagger after being aggressed. Early suspicious of intracranial damage and surgical removal of the foreign body are necessary to prevent potential complications [13–15].

## 2. Case Presentation

**Figure 1:**
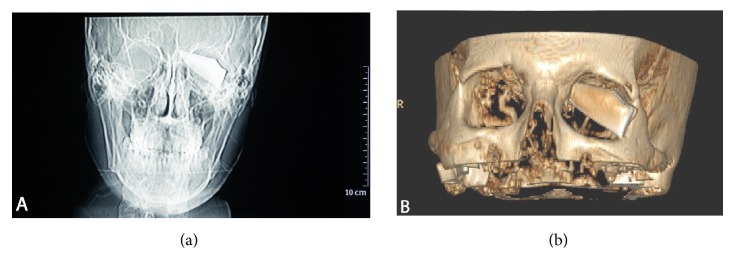
(a) Cranial X-ray showed a fragment of a dagger located intraorbitally in the superior and nasal quadrant and intracranially in the ethmoidal sinus in left site (including ruler for measurement). (b) The CT scans showed an intraorbital and intracranial foreign body of 4.6 cm x 2 cm in the left site.

**Figure 2:**
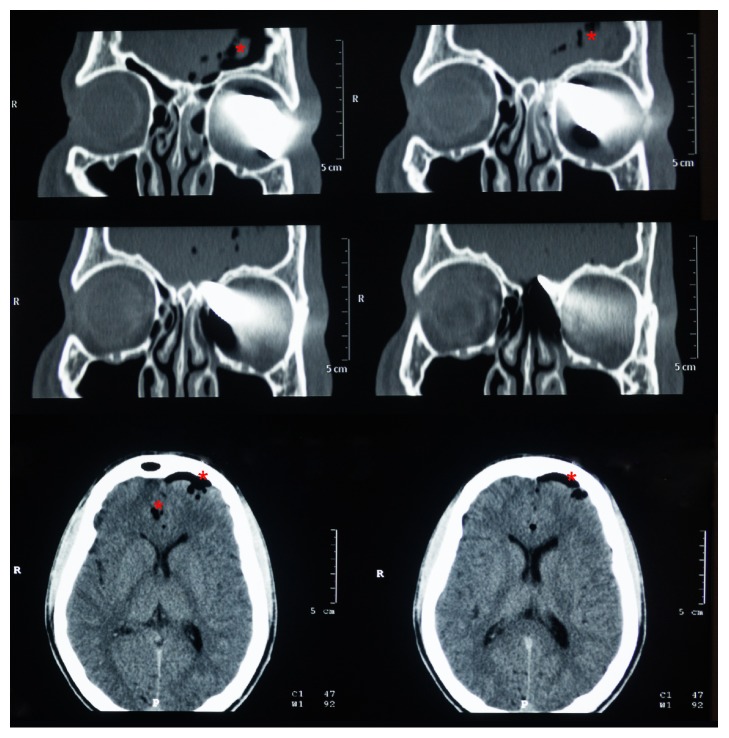
CT scans showing intraorbital foreign body with triangular shape of 4.6 cm x 2 cm in the left orbit, passing through the orbit and the ocular globe, fracturing the superomedial wall of the orbit, with a probable associated fracture of the ethmoidal cells, and reaching the anterior cranial fossa, causing pneumocephalus (marked by asterisks).

**Figure 3:**
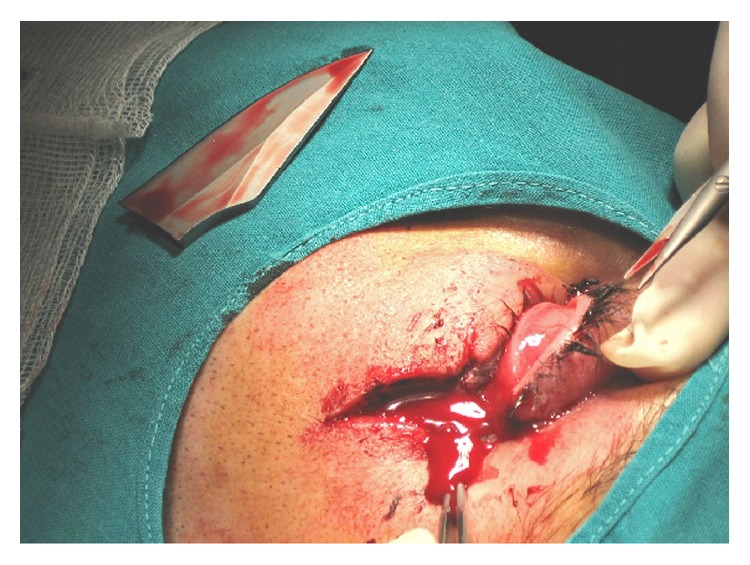
Disinsertion of the lower eyelid. The foreign body turned out to be a fragment of a dagger.

**Figure 4:**
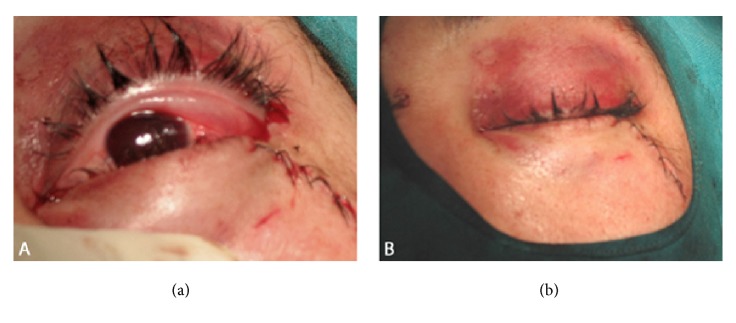
(a) and (b) Result in the immediate postoperative period is shown. The eyeball had a normal tone and the upper eyelid had a good aesthetic appearance.

A 25-year-old male patient presented to emergency department after an assault with an incise wound in the external canthus of the left eye and severe alcohol intoxication, which prevented history taking. The physical exam of the ocular globe was not possible due to the large hematoma that hindered opening the eyelid. The exploration of right eye was normal. Therefore, the skin wound was initially sutured with a polypropylene 5/0 interrupted suture and an orbital computed tomography (CT scan) and skull X-ray were performed. An intraorbital foreign body with triangular shape of 4.6 cm x 2 cm was seen in the left orbit, passing through the orbit and the ocular globe, fracturing the superomedial wall of the orbit, with a probable associated fracture of the ethmoidal cells, and reaching the anterior cranial fossa, causing pneumocephalus (Figures 1 and 2). The patient did not present any neurological symptoms beyond his alcohol intoxication nor did he develop rhinorrhea at any time, and the Glasgow Coma Scale/Score was normal (15/15). He remained under observation and was treated with intravenous antibiotic (ciprofloxacin 200 mg twice daily, for five days, selected due to its broad spectrum for gram negative and positive) and corticosteroids (methylprednisolone 80 mg per day for 3 days). Surgical extraction was performed. The foreign body turned out to be a fragment of a dagger. The extraction was done locating the end of the foreign body after removing the suture of the wound and disinserting the lower eyelid to have a wider surgical field. The foreign boy was carefully extracted without exerting force. It was then possible to see a corneoscleral wound 2 cm long affecting the upper cornea 7 mm and the sclera 8 mm located from 9 to 2 o'clock positions. It was closed with nylon 10/0 suture and polyglactin 910 7/0 suture. The entrance area in the orbit was revised, with special attention to the upper nasal quadrant, ruling out the need for repair by neurosurgery (Figure 3). One week after the surgery, the cornea was transparent, but there were amaurosis, hemophthalmos, and hypotony (Figure 4). The patient remained painless. Evolution to phthisis bulbi was evident, with clouding and folds in the cornea, shrinkage of the eyeball and a very soft tone, and six months later the eye was eviscerated. Two years later there were no signs of sympathetic ophthalmia in the right eye, whose examination remained completely normal.

## 3. Discussion

Penetrating orbitocranial injuries by nonmissile low velocity particles are quite uncommon in the literature [16]. Traumatic eye injuries due to large retained foreign bodies are even more unusual with only a few cases of a retained knife affecting the orbit [3–11]. Only one case of an intraorbital retained knife undetected during external examination and diagnosed by image tests has been reported [3]. The visual prognosis is often poor in these cases because of severe ocular damage. Suspicion is critical in the diagnosis of a hidden foreign body. In such cases the diagnosis may be delayed until complications develop weeks or months later [2, 3, 7, 8, 12].

In penetrating orbital injuries it is mandatory to rule out associated intracranial problems. The frontal lobe is the one most commonly affected in orbitocranial injuries; in most cases the foreign body will penetrate over and vertically through the orbital roof. This can be explained because most patients extend their head backwards during injury [1]. In this case, the foreign body turned out to be a fragment of a dagger, as suggested by CT scan and X-ray images (Figure 2). The fracture of the steel could have been caused by the force of the aggressor in the attack and the leverage against the outer orbital rim after the distal end of the dagger became encased in the upper nasal quadrant of the orbit.

In the treatment, hemodynamic stabilization and treatment of neurologic lesions are a priority. With regard to the eye, the initial steps are directed toward removing the foreign body and repairing the ocular damage, although both may be difficult and will not improve vision. In the current case, the removal of the foreign body caused a massive escape of intraocular contents; nevertheless, surgical reconstruction was performed. The optimal management is controversial, but evisceration seems to be an effective and safe procedure with a low risk of sympathetic ophthalmia. The prognosis of such trauma can be quite promising in cases where there are no serious complications.

In conclusion, when ruling out the possibility of an intraorbital foreign body, it is essential to perform image tests. Most foreign bodies, with the exception of wood, are detected by orbital CT scan. In our case, external signs did not suggest the presence of a foreign body, making the orbital CT scan the key tool in the diagnosis. Suspicion is critical in the diagnosis of a hidden foreign body.
